# Matrix Background Screening of an ssDNA Aptamer and Its Identification Against Lactopontin

**DOI:** 10.3390/ijms252111832

**Published:** 2024-11-04

**Authors:** Chao Zhu, Ziru Feng, Mengmeng Yan, Hongxia Du, Tengfei Li, Jiangsheng Mao

**Affiliations:** 1 Institute of Quality Standard and Testing Technology for Agro-Products, Shandong Academy of Agricultural Sciences, Jinan 250100, China; 2 College of Life Sciences and Food Engineering, Hebei University of Engineering, Handan 056038, China

**Keywords:** aptamer, SELEX, lactopontin, qPCR, dairy products

## Abstract

Lactopontin (LPN) is a highly phosphorylated O-glycosylated acidic protein closely associated with infant gut, brain, and immune development, and its recognition is urgent due to its rising application in fortified dairy products and infant formula. In this study, an ssDNA aptamer against LPN was obtained, among which two kinds of matrix-background-assisted systematic evolution of ligands via exponential enrichment (SELEX) approaches were performed and compared. The direct approach was to utilize the sample matrix as the mixing-incubation background between the ssDNA library and LPN that can theoretically increase screening pressure and simulate practical application scenarios. The indirect approach was to utilize a PBS buffer as a screening background and to include counter-screening steps that adopt the “sample matrix” as a whole as the counter-screening target. Their screening evolutions were monitored through qPCR assays from sequence diversity convergences of each sub-library based on the change in the proportion of hetero- and homo-duplexes from the dissociation curve and melting temperature, which were also verified from the sequence statistics of high-throughput sequencing. The common sequence of Seq.I1II3 from the two approaches was finally fished out as the aptamer through multiple analyses of combining the sequence frequency, secondary structures, homology, and binding assessments, which was demonstrated good specificity and low-nanomolar affinity by qPCR assay (K_D_, 5.9 nM). In addition, molecular docking and a dynamics simulation were performed for their binding site prediction and affinity confirmation. This study provides a potential identifying element and a basis for accelerating the development of methods for LPN detection in dairy products.

## 1. Introduction

Lactopontin (LPN) is a highly phosphorylated acidic protein with O-glycosylation modifications and a molecular weight of about 60 kDa, which is abundant in mammalian milk [1,2]. LPN belongs to the milk-derived osteopontin protein that was initially identified in the extracellular matrix of bovine bone [3], and its elaboration in milk began with the casein fraction of breast milk in 1987 [4], as well as being extracted from bovine milk in 1993 through crosslinked dextran gel chromatography and Q-agarose anion-exchange chromatography [5]. Until now, LPN has been extensively studied and has demonstrated a wealth of functions in both immunomodulation and the growth of neonates, infants, and young children, such as relieving inflammatory bowel diseases [6], regulating immune development [7], and affecting brain development and cognitive function [8]. For example, LPN can interact directly with invading pathogens, bind to α_x_β_2_ integrin receptors, induce IL-1 activation of monocyte migration, and enhance phagocytosis [9]. In general, the concentration of LPN in breast milk ranges from 48 to 266 mg/L, which is much higher than that in cow’s milk (about 18 mg/L) and infant formula (about 9 mg/L) [10], which makes LPN considered a potential candidate functional protein ingredient for use by infant formula manufacturers to complement their formulas for reducing the difference in concentration between breast milk and cow’s milk and make their formulas breastfeeding-friendly for infants and young children [11]. In 2023, Arla Foods Ingredients’ application to place cow’s milk LPN on the European market in infant formula and milk drinks with a maximum allowable use of 151 mg/L was approved and adopted by the European Food Safety Authority [12]. Due to its efficacy [13,14], LPN-containing infant formulas are highly sought after by consumers, especially for non-breastfeeding families. Although many products on the market already claim to contain or add LPN, few relevant National Standards for LPN determination in dairy products have been established.

Currently, two analytical approaches, liquid chromatography coupled with mass spectrometry (LC-MS) as the main detection approach and enzyme-linked immunosorbent assay (ELISA) as the main recognition approach, are generally employed to determine the presence of LPN in dairy products. For example, Hu et al. developed an ultrahigh performance LC-MS/MS method and achieved LPN determination in bovine, buffalo, yak, sheep, and goat milk with a good limit of detection of 0.5 mg/L [15]. However, LC-MS-based methods generally have difficulty quantifying the intact LPN directly and are usually based on a signature peptide [15,16,17]. In addition, a complex sample preparation procedure is typically needed, including trypsin digestion, dimethyl labeling of tryptic peptides, purification, and the concentration of labeled tryptic peptides with solid-phase extraction [17]. Apart from LC-MS, ion-exchange chromatography (e.g., anion-exchange chromatography) [18], reversed-phase HPLC [19], and non-gel sieving capillary electrophoresis [20] are also getting a lot of attention. Although chromatographic-based methods are typically traditional and effective methodologies, their application can be negatively affected by some aspects, including inconvenient portability and the requirement for expensive instruments and trained professionals. For the complete and independent identification of LPN, the antibody-based ELISA has been extensively incorporated into convenient products because of its easy procedures [1]. There are general commercial monoclonal antibodies to human, mouse, or rat osteopontin, but some of them cross-react with bovine LPN, which leads to overestimated results [21]. In addition, the discovery of antibodies generally involves long cycles of animal immunization experiments, as well as stringent conditions to guarantee their stability [22]. Therefore, it is important to seek other molecular recognition candidates against LPN.

Nucleic acid aptamers (aptamers) are a class of oligonucleotide sequences (ssDNA or RNA) that can specifically recognize a wide range of targets (e.g., small molecules, proteins, cells, bacteria, and tissue sections) with high affinity, typically screened via the recognized strategy of systematic evolution of ligands by exponential enrichment (SELEX) [23]. They have been studied extensively since 1990 [24,25] and are seen as an appealing alternative to antibodies, which has led to the term “chemical antibody”, and they can compensate for the weaknesses of antibodies [26]. They are easily produced by standard oligonucleotide synthesis approaches, eliminating additional chemical linkage procedures; they are more stable, have batch-to-batch consistency, have fewer limitations in their targets, and have low immunogenicity, which has resulted in them attracting wide attention in many fields [27,28,29]. There have been two aptamer drugs approved by the FDA: Pegaptanib (Macugen) [30] and Zuranolone (Zurzuvae) [31]. As of 2024, over 1400 aptamer entries are accessible in the aptamer [32], and more than 30 SELEX methods and their derived strategies have been reported to improve aptamer screening efficiency [33,34,35,36]; however, the number of aptamers actually applied or productized is very limited, and even some aptamer experiments are not reproducible [37]. An important reason for this is the difference between the screening conditions and the real application environment, in which complex matrix effects or environmental factors may interfere with the spatial conformation of the aptamers and thus affect the binding of aptamers to targets [38]. However, only a handful of real-sample-assisted approaches have been employed for screening aptamers, such as fetal sheep serum as the screening solution [39] and a molecular crowding approach strategy [40] from a thermodynamics point of view. Therefore, there is still room for investigating the efficiency of real-sample-assisted SELEX. Also, there are no reports on aptamer screening against LPN in dairy products.

In the current work, we employed matrix background SELEX for the discovery of an ssDNA aptamer for LPN, in which two kinds of screening approaches were performed and compared: the direct one was to utilize a milk powder matrix as the mixing-incubation background between the ssDNA library and LFN, and the indirect one was to utilize a “sample matrix” as a whole as the counter-screening target. A dissociation curve analysis in qPCR was freshly employed to monitor the screening evolution through the diversity change in each sub-library regarding the proportion of hetero- and homo-duplexes, which was also verified via the high-throughput sequencing (HTS) results. Through multiple analyses of combining the sequence frequency, secondary structures, homology, and binding assessments, the high-affinity sequence of Seq.I1II3 was finally fished out from these two approaches as an aptamer with a low-nanomolar dissociation constant (K_D_, 5.9 nM) determined by qPCR and with good selectivity. In addition, a molecular dynamics (MD) simulation was performed for the prediction of binding sites and confirmation of affinity. This aptamer provides a potential identifying element and a basis for the development of methods for the detection of LPN in dairy products.

## 2. Results and Discussion

### 2.1. Illustration of Aptamer Screening

**Figure 1:**
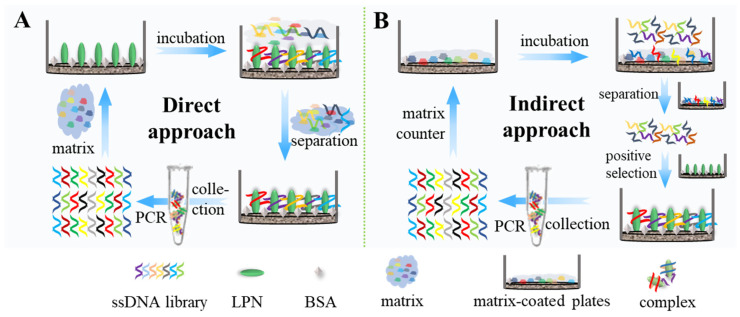
Schematic illustration of the direct (**A**) and indirect (**B**) matrix background SELEX approaches.

Figure 1 illustrates the principle of the two approaches of matrix background SELEX for the ssDNA aptamer. Since the sequence diversity in the initial ssDNA library is high and the proportion of sequences with affinity or high affinity for the target might be low, in the first round of screening, a more benign environment of PBS buffer was adopted for rapidly enriching the affinity of the ssDNA sequences. The enriched second sub-library was then divided equally into two portions, and matrix background screening was carried out for each portion in a separate approach. The direct approach (Figure 1A) was to utilize the sample matrix to dilute the ssDNA library as the mixing-incubation background, which can theoretically both increase screening pressure and simulate practical application scenarios. The indirect approach (Figure 1B) was to utilize PBS buffer as a screening background and include a counter-screening step, in which the “sample matrix” as a whole was adopted as the counter-screening target since a single component is often used as the counter-screening target in conventional screening; however, the actual samples are often very complex, and analyzing them one by one to achieve all the counter-screening components is in fact unrealistic. Prior to each round of screening, the ssDNA library was initially added into the prepared bovine serum albumin (BSA)-coated microplate that enables the protein to passively absorb through a hydrophilic surface of MaxiSorp for performing negative screening that is aimed to remove ssDNA sequences that are non-specifically adsorbed to the microplate or BSA. Subsequently, the retained free solution containing available ssDNA sequences that are not involved in binding was gently pipetted into an empty target-protein-coated and BSA-blocked microplate for positive screening. After their mixing incubation, recognition, and washing, the complex of the “Target/ssDNA” was yielded and retained in substrate wells and then eluted down through a simple heating process, followed by being subjected to symmetric/asymmetric PCR amplification to yield a new sub-library applied in the next screening. During screening rounds, the sequence diversity convergence of each sub-library was compared through quantitative real-time PCR (qPCR) to readily monitor the screening progress. The determined affinity-enriched sub-library of each separate approach was subjected to high-throughput sequencing (HTS) on the Illumina-Miseq platform, and the parent candidate sequences were hand-picked through multiple analyses of combining homology and frequency, in addition to their secondary structures. The binding affinity of the finalized sequence was characterized by a qPCR assay, as was its specificity performance. The strategy of matrix background SELEX for the ssDNA aptamer illustrates the feasibility of this approach and that it can be recommended as a general screening method.

### 2.2. Matrix Background SELEX for LPN

To conduct the feasibility of this matrix background SELEX, the lactopontin (LPN) protein was chosen as the model protein, followed by a screening workflow for forming the complex of “LPN/ssDNA”. The clear band (Figure S1) in the agarose gel electrophoresis (AGE), band 1, represents the dsDNA product of the complex after the first screening round, which was presented at the right position of about 80 bp, whereas band 2 was the blank control with no clear band. This suggested a positive result of the screening. After the first round of screening, the milk powder matrix was utilized to dilute the ssDNA library in the direct approach as a mixing-incubation background, as well as used with a gradual decrease in substrate dilution progressively with increasing screening rounds (10× in the second, 5× in the third, and 2× in the fourth, sixth, eighth, and tenth). This screening workflow does not incorporate a counter-screening step but simply intersperses a PBS-only background in the relevant rounds (fifth, seventh, and ninth) for better purity of the obtained sequences. Differently, PBS buffer was used as the screening solution in the indirect approach, which also introduces the counter-screening step separately after the third, fifth, seventh, and ninth rounds, wherein the “milk powder matrix” as a whole was used as the counter-screening target for indirectly providing the matrix background.

**Figure 2:**
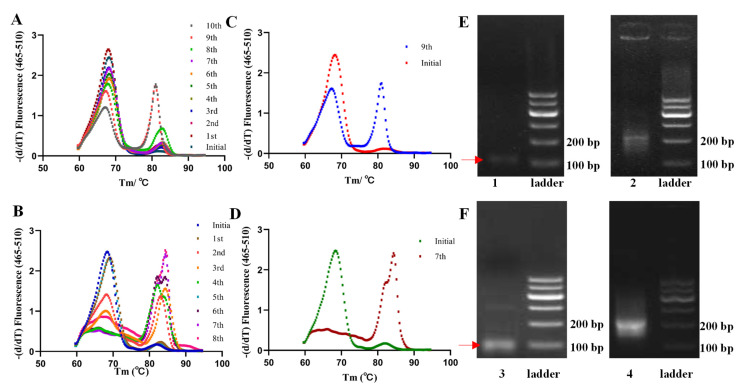
The dissociation curve analysis for each library to monitor the screening evolution: ten rounds (**A**) and the clear curves’ changes (**C**) in the direct approach; eight rounds (**B**) and the clear curves’ changes (**D**) in the indirect approach; their separate AGE results (**E**,**F**) of the PCR products after linking the connectors before HTS changed from 80 bp (bands 1 and 3) to the position of 200 bp (bands 2 and 4).

Most previous studies describing the screening of aptamers against proteins applied spectrophotometric measurements (e.g., SPR) to determine and monitor the proportion of ssDNA bound to the protein and terminated the screening once a plateau was reached. Herein, qPCR was employed to monitor the screening evolution for each library in these two approaches through their dissociation curve analysis, which enables the qualitative assessment of the diversity change of these LPN-bound ssDNA sequences, wherein the ssDNA diversity determines the proportion of hetero- and homo-duplexes during PCR, i.e., the higher the diversity, the greater the proportion of hetero-duplexes, in turn, responding to lower melting temperature (Tm) changes compared to homo-duplexes. As is evident from Figure 2A,B, the initial library owing the most diversity presented a main melting peak with an average Tm of approximately 70 °C, and the latter sub-libraries observed distinct melting peaks with a shift towards a higher Tm that was attributed to a change in pool composition and the accumulation of ssDNA. In the direct (Figure 2C) and indirect (Figure 2D) approaches, we observed that distinct melting peaks appeared in the ninth sub-library, and the melting peak increased almost completely to a higher Tm of approximately 86 °C without distinct melting peaks in the seventh. In addition, there were no more visible changes in the further rounds, which all suggests a drop in pool diversity and the emergence of potentially enriched sequences in these two approaches. Clearly, the sequence diversity in the enriched ninth sub-library is higher than that in the seventh sub-library, which could also be explained by that although the direct approach using a sample matrix as a screening background increases the screening pressure, this complex matrix is somewhat difficult for ssDNA enrichment and requires more rounds. These two approaches were ended separately at the seventh and ninth rounds, as further rounds are not only costly and time-consuming but can also make the screening prone to amplification errors or even the loss of affinity sequences. Therefore, the affinity-enriched libraries were subjected to high-throughput sequencing (HTS) on the Illumina-Miseq platform.

### 2.3. Comprehensive Evaluation of Candidate Sequences

**Figure 3:**
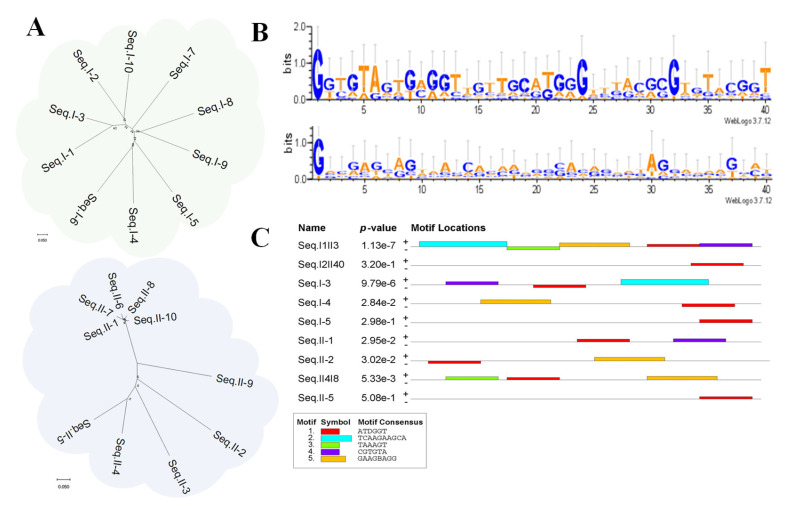
(**A**): Evolutionary tree of the top 10 sequences of the direct approach (top) and indirect approach (bottom) via MEGA-11. (**B**): Their sequence logos of conserved bases through a Clustal X analysis of the direct approach (Top) and indirect approach (bottom). (**C**): The motif analysis of the selected nine candidate sequences through MEME Suite.

Prior to the HTS, the dsDNA concentrations in the ninth and seventh libraries were separately determined to be 21.0 ng/μL and 33.8 ng/μL after symmetric PCR amplification, which met the subsequent HTS requirements. These dsDNA concentrations were then linked to the sequencing connectors (120 bp) through symmetric PCR amplification again based on the molecular-tag-containing primers. From the AGE in Figure 2E,F, the band positions of the PCR products after linking the connectors changed from 120 bp to the position of 200 bp, suggesting successful linkage. Following HTS, the total sequence of 75,240 with a higher diversity of 45,010 in the direct approach was obtained, while a similar total sequence of 71,509 was obtained in the indirect approach; however, it had a much lower diversity of 14,128, results that also verified the change in sequence diversity in the dissociation curve analysis (Figure 2C,D). In terms of the filtering frequency analysis, only 14 sequences obtained by the direct approach have a frequency greater than 100, while there are more than 50 such sequences in the indirect approach. In addition, comparing the top 10 sequences (Table S1), the frequency of the sequences obtained by the direct approach are all much lower than the frequency of the sequences obtained by the indirect approach, even by an order of magnitude. Also, the top 10 sequences from the two approaches were separately subjected to a sequence homology analysis (Figure 3A) by developing evolutionary trees via MEGA-11 software [41], and the tree branches obtained by the indirect method are more clustered, while those obtained by the direct method are dispersed, which was verified through the online application WeLogo3 to generate a sequence logo with the accumulation of residues at each position reflecting their conservation degree (Figure 3B). All these results also confirm that the sequences obtained by the indirect approach are more convergent.

However, a notable comparison of the top ten sequences in terms of the overlap in the two fasta files reveals that of the ten sequences with high frequency in the indirect approach, only two appear in the direct approach, but the top nine sequences in the direct approach are detected in the indirect approach (Table S1), where sequences with a frequency of 1 are not considered to meet the enrichment definition and are therefore not included in the statistics. Especially, the two most highly enriched sequences (27,306 and 2683) in the indirect approach were not suitable for enrichment in the direct approach, i.e., they were not suitable for binding to LPN in the matrix environment, but instead, their sequences Seq.II-3 and Seq.II-40 became two highly enriched sequences in the direct approach, Seq.I-1 and Seq.I-2 (Table 1). These comparisons illustrated that the sequences enriched by the indirect approach do not necessarily result in stronger binding to the target under practical conditions.

### 2.4. Performance Evaluation of the Candidate Aptamer

**Figure 4:**
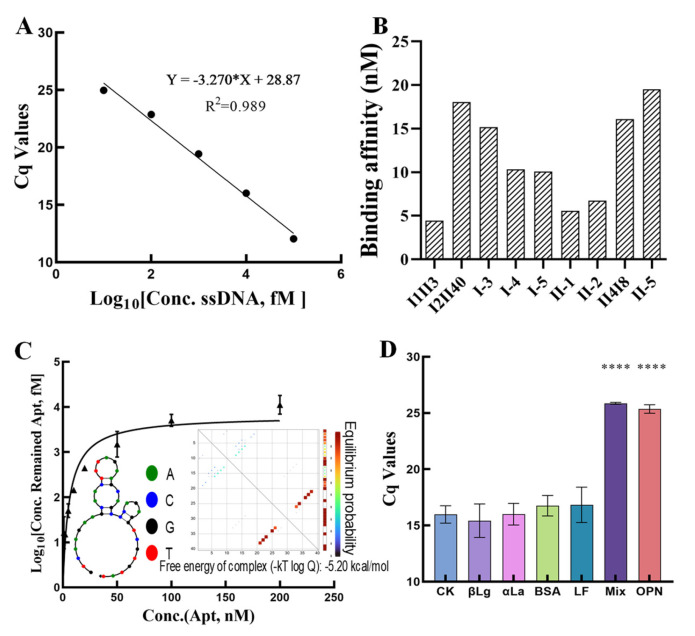
(**A**): The ssDNA standard solutions through qPCR assay between ssDNA concentration and Cq values. (**B**): The preliminary affinity evaluation of the nine selected candidate sequences. (**C**): The affinity of Seq.I1II3 with seven concentration (Conc.) levels (0, 5, 10, 20, 50, 100, and 200 nM) based on the non-linear fitting method. (**D**): The specificity assessment of Seq.I1II3 (**** *p* < 0.0001).

The first five sequences with high frequency from each approach containing three repeating sequences were evaluated for binding to LPN (Table 1), wherein I-# represents the sequences obtained in the direct approach and II-# represents the sequences obtained in the indirect approach, while I#II# represents the sequences in both two approaches. To characterize their affinity, a novel method was developed based on the parameter of threshold cycle (Cq) in qPCR, where the Cq is the number of amplification cycles elapsed during qPCR amplification when the fluorescence signal of the amplified product reaches a set threshold that is linearly related to the logarithm of the starting template concentration. First, five concentration levels (0.01, 0.1, 1, 10, and 100 nM) of ssDNA standard solutions were subjected to qPCR assay, and a standard curve was established (Figure 4A) by using the logarithmic value of the ssDNA concentration as the horizontal coordinate and the Cq value corresponding to each concentration as the vertical coordinate with a linear equation of y = −3.270x + 28.87 (R^2^, 0.989). Subsequently, the direct recognition assay in the LPN-coated microplate was carried out through three steps: (i) adding different concentrations of candidate sequences into the LPN-coated microplate followed by incubation; (ii) washing, re-dissolution, and heating; (iii) performing qPCR on the retained sequence solution. Through their Cq values and the established standard curve, the binding (retained) sequence concentrations were derived. Based on the non-linear fitting method using the logarithmic value of the binding (retained) sequence concentrations as the vertical coordinate and the adding sequence concentrations as the horizontal coordinate, preliminary evaluation was performed on the total sequences from three concentration levels (Table S2 and Figure 4B), and Seq.I1II3 presented the best affinity. In detail, Seq.I1II3 with seven concentration levels (0, 5, 10, 20, 50, 100, and 200 nM) was accurately determined at a nanomolar magnitude affinity (K_D_, 5.9 nM) against LPN based on the non-linear fitting method (Figure 4C), suggesting the high-efficiency screening of matrix background SELEX. Also, the nine selected sequences that may correspond to the protein-binding site were subjected to a motif (Figure 3C) analysis through MEME Suite [42]. Figure 3C shows the five discovered motifs and displays their motif locations. According to the combined match *p*-values (Table 1), which is defined as the probability that a sequence would have position *p*-value such that the product is smaller or equal to the value calculated for the sequence under testing, there is a lower *p*-value of 1.13 × 10^−7^ in Seq.I1II3. These results indicated that Seq.I1II3 was more likely to have superior affinity compared to the other sequences. Consequently, Seq. I1II3, with the best affinity, was selected as the aptamer sequence.

To verify the binding specificity of Seq.I1II3 for LPN (200 nM), a competition recognition assay in the LPN-coated microplate assay, which differs from the direct recognition assay in that Seq.I1II3 is first incubated with the protein before being added to the microplate, was performed. It was performed on other popular proteins in whey, including α-La and β-Lg, the coated BSA proteins, and LF, which can form complexes with LPN separately with a high concentration of 2 μM, and their protein mixtures. Figure 4D shows that the Cq values of LPN and Mix were higher than those of several other proteins (*p* < 0.0001) even with a tenfold difference in concentration. These results all indicated Seq.I1II3’s high specificity towards LPN and that these proteins and their mixtures do not affect the recognition of LPN by this aptamer.

### 2.5. Binding Analysis by Molecular Dynamics

**Figure 5:**
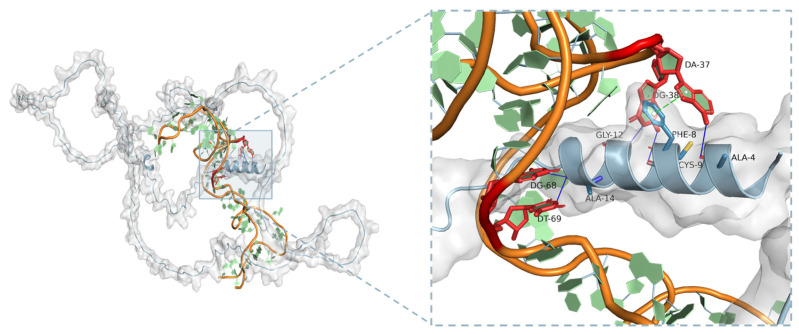
Binding mode analysis between the aptamer and LPN. The accompanying figure delineates the specific binding site with amino acid residues represented as blue stick models and nucleotide residues as red stick models.

Molecular dynamics (MD), employed alongside wet experiments, is a powerful method used to predict or explore the interaction between ligands and receptors [43]. The structure of LPN was obtained from the AlphaFold Protein Structure Database (UniProtP: 31,096), and none is available in the PDB. The secondary structure of Seq.I1II3 was predicted using M-fold (www.unafold.org/mfold/, ΔG = −3.34 kcal/mol). With the help of PyMOL software (Version 3.11), interactions between the aptamer Seq.I1II3 and LPN were simulated to find the optimal docking model selected based on the binding free energy. This is the first time MD has been employed to predict the recognition between the aptamer and LPN. The 3D structure is shown in Figure 5, which was thought to be the best docking result. Based on the docking model, three different interactions were observed between Seq.I1II3 and LPN, including five hydrogen bonds, one hydrophobic interaction, and one Pi-Pi bond. Associating Figure 5 and Table S3, the binding sites in Seq.I1II3 involved four main nucleotides of A37, G38, G68, and T69. This indicated that the conserved motifs in Seq.I1II3 were likely to bind to the important structural domains in LPN proteins. The MD analysis can give some clues to further explore the aptamer interaction mechanism.

## 3. Material and Methods

### 3.1. Materials, Reagents, and Instruments

A MaxiSorp-surfaced 96-well microplate was purchased from Thermo Fisher Scientific (Waltham MA, USA). NaCl, KCl, H_3_PO_4_, Na_2_HPO_4_, NaH_2_PO_4_, Na_2_B_4_O_7_·10H_2_O, NaHCO_3_, Na_2_CO_3_, NaOH, H_3_BO_3_, CH_3_COOH, HCl, H_2_SO_4_, Tween-20, anhydrous ethanol, trichloromethane, isoamyl alcohol, and isopropyl alcohol, all analytically pure, were purchased from Sinopharm Chemical Reagent Co., Ltd. (Beijing, China). Bovine lactoferrin (Lf, purity ≥ 85%, SDS-PAGE), α-lactalbumin (α-La, from cow’s milk, purity ≥ 85%), bovine serum albumin (BSA, purity ≥ 98%), β-lactoglobulin (β-Lg, from cow’s milk, purity ≥ 90%), and lactopontin (LPN, from cow’s milk, purity ≥ 90%, SDS-PAGE) were purchased from Sigma-Aldrich LLC (Beijing, China). Agarose (Biowest), 2×Taq PCR premix reagent (without dye), sterile and enzyme-free deionized water, GeneGreen nucleic acid dye, 6 × DNA loading buffer (bromophenol and xylene cyan), 50 bp dsDNA ladder, and 5 × TBE Buffer were purchased from Tiangen Biotech Co., Ltd. (Beijing, China). Hieff UNICON Universal Blue qPCR SYBR Green Master Mix was purchased from Yeasen Biotechnology Co., Ltd. (Shanghai, China). Primer sequences (forward 5′-AGCAGCACAGAGGTCAGATG-3′, reverse 5′-TTCACGGTAGCACGCATAGG-3′), the random ssDNA library (80 nt, 5′-AGCAGCACAGAGGTCAGATG-N_40_-CCTATGCGTGCTACCGTGAA-3′), and the selected candidate sequences were all ordered and purified by Shanghai Sangon Biotechnology Co., Ltd. (Shanghai, China).

The instrument of LightCycler^®^ 480 II (Roche, Switzerland) was employed to perform the real-time fluorescence quantitative PCR assay. A GENESY 96T Cycler PCR device (Xi’an TianLong Science and Technology Co., Ltd., Xi’an, China) was used for PCR amplification experiments. The experimental water was ultrapure and purified by a Milli-Q system (resistivity of 18.2 MΩ-cm@25 °C) (Millipore, Bedford, MA, USA). The nucleic acid fragment was confirmed by a T-green transilluminator (Tiangen Biotech, Beijing, China). Next-generation sequencing (Miseq platform, Illumina) was supplied by Shanghai Sangon Biotechnology Co., Ltd. (Shanghai, China). A PowerPac™ Basic Power Supply (Bio-Rad, Hercules, CA, USA) was utilized to perform the agarose gel electrophoresis (AGE) assay.

### 3.2. Buffer Preparation

PBS buffer preparation was performed according to the following steps: 0.24 g NaH_2_PO_4_, 1.42 g Na_2_HPO_4_, 7.89 g NaCl, and 10.06 g KCl were weighed in a beaker, 900 mL of ultrapure water was added, HCl was used to adjust the pH to 7.3 ± 0.1, and the mixture was transferred to a 1 L volumetric flask and shaken well. A PBST solution (PBS buffer containing 0.05% Tween 20) was prepared as follows: 400 mL of PBS buffer was taken and 200 μL of Tween 20 was added; it was shaken well and set aside. Carbonate (CBS, 0.1 M) buffer preparation was performed according to the following steps: 8.40 g NaHCO_3_ and 10.60 g Na_2_CO_3_ were weighed, respectively, and volume was increased to 500 mL with ultrapure water to obtain 0.2 M mother liquor. Then, 16 mL of 0.2 M NaCO_3_ and 34 mL of 0.2 M NaHCO_3_ were taken, and they were mixed thoroughly. An acetic acid solution (50 mM) was prepared as follows: 286 μL of glacial acetic acid was taken, and the volume was fixed to 100 mL with ultrapure water. The preparation of the milk powder matrix solution was as follows: 50 mg of milk powder was weighed in a 1.5 mL centrifuge tube; 100 μL of ultrapure water was added to make it fully dissolved, and then 900 μL of the 50 mmol/L acetic acid solution was added. The solution was vortexed and mixed well, left for 10 min, and then centrifuged for 10 min at 8000 r/min. Then, the intermediate clear liquid layer was taken through a 0.22 μm cellulose acetate membrane, it was diluted by 1-fold with PBS, and then stored at 4 °C. Configuration of 0.5 × TBE buffer was performed as follows: 100 mL of 5 × TBE Buffer was measured in a reagent bottle, 900 mL of ultrapure water was added, and it was mixed thoroughly at room temperature to formulate the 0.5 × TBE Buffer. The ssDNA solution preparation was performed including the ssDNA library, forward and reverse primers, aptamer sequences, etc. Firstly, the ssDNA tube was centrifuged at 4000 r/min for 60 s. Thereafter, the corresponding volume of double-distilled water was added following the volume shown on the label of the ssDNA tube, and it was vortexed for 30 s to obtain an ssDNA solution of 100 μM. The sample solution was stored at 4 °C for spare parts and stored at −20 °C long term. Agarose gel preparation was performed as follows: 2.4 g (4% concentration) of agarose was weighed in a conical flask; 60 mL of 0.5 × TBE buffer was slowly added, it was mixed well, 1.2 μL of GeneGreen nucleic acid dye was added, and it was mixed again. Then, the mixture was heated to boiling, then briefly cooled and heated again to boiling. This was repeated 3 times until the solution was a uniform and transparent gel, and when it cooled to 60 °C or so, it was poured into a gel-making tank. Different-sized combs were inserted as needed, and it was let cool for 40 min before use.

### 3.3. Screening Process

An amount of 100 μL of the LPN solution (5 μg/mL) was added to the blank 96-well plate and coated at 4 °C for 12 h. After that, the solution in the wells was poured out and washed with PBS-T 3 times and patted dry. Then, 200 μL of the BSA solution (1%) was added into each well, followed by 60 min of incubation, and the solution in the wells was poured out and washed with PBS-T 3 times. To avoid screening for sequences that adsorb non-specifically to the walls of the plate and BSA, 100 μL of the ssDNA library (2 μmol/L) was added to only the BSA-coated microplate wells that did not contain LPN before the start of each round for 30 min of negative screening, and the retained free solution containing available ssDNA sequences that are not involved in binding was gently pipetted into an empty LPN-coated and BSA-blocked microplate for positive screening for 30 min and then washed with the PBST solution again. Then, 100 μL of ddH_2_O was added to the wells, which were sealed and treated at 80 °C for 10 min, and the liquid in the wells as complexes was aspirated for amplification. After the first screening round, the second sub-library was then divided equally into two portions, and matrix background screening was carried out for each portion in a separate approach. The direct approach is to utilize the milk powder matrix to dilute the ssDNA library as a mixing-incubation background, as well as to use a gradual decrease in the substrate dilution progressively with increasing screening rounds (10× in the second, 5× in the third, and 2× in the fourth, sixth, eighth, and tenth) and intersperse the PBS-only background in the relevant rounds (fifth, seventh, and ninth). The indirect approach is to utilize PBS buffer as a screening background and to include a counter-screening step separately after the third, fifth, seventh, and ninth rounds using the “milk powder matrix” as a whole adopted as the counter-screening target. The rest of the steps remained unchanged. 

### 3.4. PCR Amplification and ssDNA Purification

The collected complex first underwent symmetric PCR, which was carried out in a reaction system (sample, 2 μL; forward and reverse primers, added separately 10 μL of 10 μM; Mix enzyme, 20 μL; ddH_2_O, 8 μL). The Mix enzyme consisted of 0.1 U Taq Polymerase/μL, 500 μM dNTP, 20 mM Tris-HCl (pH 8.3), 100 mM KCl, and 3 mM MgCl_2_. The cycling parameters for the PCR amplification are single denaturation at 94 °C for 1 min, 20 cycles at 94 °C for 30 s, 55 °C for 30 s, and 72 °C for 30 s. Then, asymmetric PCR was performed to obtain ssDNA, in which the differences compared to symmetric PCR were that the concentration of the reverse primer decreased at 84 nM and 30 cycles of amplification rounds. The length and purity of PCR products were verified through agarose gel electrophoresis (AGE) analysis (4.0% agarose, 30 min, 160 V). The target strip was cut with a scalpel. The purification procedures are as follows. A 300 mg agarose gel containing the target bands was put into a 2 mL centrifuge tube and frozen at −20 °C for 1 h before being broken. A total of 500 μL of distilled water was added to the tube and vortexed for 60 s. A total of 500 μL of DNA-extracting phenol reagent (mainly tris-saturated phenol with 8-hydroxyquinoline) was added and continued to be vortexed for another 60 s. At the end of the vortexing process, the sample was centrifuged at 13,000 r/min in a refrigerated centrifuge for 15 min at 4 °C. The supernatant of the centrifuged sample was taken into a 1.5 mL centrifuge tube, the supernatant was added into a 1.5 mL centrifuge tube, and an equal volume of trichloromethane–isopentanol mixture (24:1) was added. The supernatant was placed in a 1.5 mL centrifuge tube, and an equal volume of trichloromethane–isoamyl alcohol mixture (24:1, *v*/*v*) was added; it was vortexed for 60 s and then centrifuged again under the same conditions as above. After 400 μL of the upper layer of the centrifuged liquid was put into a 1.5 mL centrifuge tube, 40 μL of the 3 mol/L sodium acetate solution and 1000 μL of pre-cooled isopropanol were added sequentially, mixed well, and then centrifuged at −20 °C for 2 h. The precipitate was washed with 70% anhydrous ethanol, centrifuged twice, and then the supernatant was discarded and dried at room temperature and re-dissolved in 50 μL of double-distilled water. It was then stored at 4 °C for spare use.

### 3.5. The Characterization of Affinity and Specificity

The screening evolution of each sub-library was determined using qPCR in these two approaches through their dissociation curve analysis. The qPCR was carried out in a reaction system (sample, 1 μL; forward and reverse primers, added separately 0.4 μL of 10 μM; qPCR SYBR Green Master Mix, 5 μL; ddH_2_O, 3.2 μL). The cycling parameters for PCR amplification were single denaturation at 95 °C for 2 min, 40 cycles at 95 °C for 10 s, and 60 °C for 30 s of annealing and extension. The preliminary binding characterizations of twelve candidate sequences were determined based on the parameter of threshold cycle (Cq) in qPCR. First, five concentration levels (0.01, 0.1, 1, 10, and 100 nM) of ssDNA standard solutions were subjected to qPCR assay, and a standard curve was established by using the logarithmic value of the ssDNA concentration as the horizontal coordinate and the Cq value corresponding to each concentration as the vertical coordinate. Subsequently, the direct recognition assay in the LPN-coated microplate was carried out through three steps: (i) adding different concentrations of twelve candidate sequences (20, 100, and 500 nM) into LPN-coated microplates followed by incubation; (ii) washing, re-dissolution, and heating; (iii) performing qPCR on the retained sequence solution. Through their Cq values and the established standard curve, the binding (retained) sequence concentrations were derived, which in turn determined the best sequences of Seq.I1II3. The affinity of Seq.I1II3 was determined using the same direct recognition assay by using more concentrations of Seq.I1II3 (0, 5, 10, 20, 50, 100, and 200 nM) and using the non-linear fitting method Y = Bmax * X/(K_D_ + X), where Y represents the average value of the binding concentrations, Bmax represents the maximum binding concentrations, X represents the added concentration of the aptamer, and K_D_ is the equilibrium dissociation constant, with the unit being the same as that of X.

The specificity evaluation of Seq.I1II3 to LPN was performed in the competition recognition assay in the LPN-coated microplate, which differs from the direct recognition assay in that Seq.I1II3 is first incubated with the protein before being added to the microplate. In the above-introduced procedures of these assays, the LPN concentration was 200 nM, and the concentration of other proteins (including α-La, β-Lg, BSA, and Lf) was 2 μM, as was the mixture of the five proteins.

### 3.6. Structural Analysis and Molecular Docking

The secondary structures of both the original and truncated candidate sequences were simulated using NUPACK and M-fold (accessed on 10 July 2024, www.unafold.org/mfold/). We utilized online tools for motif prediction and analysis to understand the role of sequence motifs in binding to target proteins. Specifically, MEME (accessed on 10 July 2024, http://meme-suite.org/) and WebLogo3 (accessed on 8 July 2024, http://weblogo.threeplusone.com/) were employed to predict motifs of candidate aptamers without primers. MEME, utilizing the maximum expectation (EM) algorithm, integrates various functions, such as motif discovery, enrichment, and comparison functions. The number of structural domains (motifs) was set to five, and the program was operated to observe the differences in the distribution of motifs in different sequences, marked with different colors. Additionally, WebLogo3 provides a graphical representation of residue frequencies at each position, facilitating motif analysis.

The molecular docking of the sandwich format between the LPN protein and the aptamer Seq.I1II3 was performed as the method reported in [44]. Briefly, the 3D structures of the LPN protein and the aptamer Seq.I1II3 were obtained from the AlphaFold Protein Structure Database (UniProtP: 31,096) and the 3dRNA/DNA tertiary structure prediction method, respectively. HNADOCK server was based on a hierarchical docking algorithm that includes an FFT-based global search strategy and an intrinsic scoring function for nucleic acid interactions. The docking model with the highest score was subjected to a non-covalent interaction analysis using the Protein-Ligand Interaction Profiler (PLIP) web tool and then visualized by PyMOL molecular visualization software (Version3.11).

## 4. Conclusions

To conclude, the aptamer (Seq.I1II3) against the LPN protein was obtained with good affinity (K_D_, 5.9 nM) and specificity by matrix background-assisted SELEX, in which the screening evolutions were monitored by a qPCR assay in conjunction with HTS statistics. By comparing the two matrix background screening methods, three insights were gained: (i) although using the sample matrix as a screening background directly increases the screening pressure, the complex matrix is somewhat difficult to use for sequence enrichment and requires more rounds; (ii) the sequences enriched by the indirect approach do not necessarily result in stronger binding to the target under practical conditions; (iii) the procedures in the direct approach are somewhat less cumbersome than those in the indirect approach. In addition, the binding sites between the aptamer and LPN were predicted through MD simulation, which would be significant for further studies to understand the aptamer function. This aptamer provides a potential identifying element and a basis for the development of more sensitive aptasensors for LPN detection. Furthermore, given the stereotypical nature of the SELEX process, the screening mindset could be more practically orientated to discover a high-performance aptamer, and a matrix background screening strategy might be more recommended.

## Figures and Tables

**Table 1:** The information of the candidate sequences, frequency, ratio, and combined match *p*-values.

Seq.	Bases Composition (5′F-3′R)	Frequency	Ratio (%)	*p*-Value
I1II3	GTCAAGAAGCAACTTTAGAAGCAGGGGAGAGGTCGTGTAT	3595 vs. 1680	4.8 vs. 2.44	1.13 × 10^−7^
I2II40	GGAGGGCAGTCAAAAACGGGCAGCACTCTAGTAAAGGTCG	627 vs. 137	0.84 vs. 0.2	3.20 × 10^−1^
I-3	GAGACGTGTATAGCACCAATTGAATCAAGAAGCAGCTACC	557	0.74	9.79 × 10^−6^
I-4	GGGCATGGGAAGTAGGGATAGGCCGGTTTCCACCAATGAG	222	0.3	2.84 × 10^−2^
I-5	GCCAATTTCCGGGCACCTGGACACAGAATACTGATAGGTT	177	0.24	2.98 × 10^−1^
II-1	GGTGTAGTGAGGTTGTTGCATGGGTTTACGCGTGTACGGT	27,306	39.65	2.95 × 10^−2^
II-2	GGATCTATGTCATCACACACGGATGGAGGAGTGCATTCGCT	2683	3.9	3.02 × 10^−2^
II4I8	GGCGTAAAGTGATCGGTACGGGAAAGGGAAGGATGCTTAT	1111	1.61	5.33 × 10^−3^
II-5	GTCAAGCGTCGGTGCCGCTCGGGGAGCCCACTAATGGATG	441	0.64	5.08 × 10^−1^

## Data Availability

Data are contained within the article and Supplementary Materials.

## Supplementary Materials

The following supporting information can be downloaded at: https://www.mdpi.com/article/10.3390/ijms252111832/s1.
