# Supplementary material for: Matrix Background Screening of an ssDNA Aptamer and Its Identification Against Lactopontin
Source: Int J Mol Sci. 2024 Nov 4;25(21):11832. doi: 10.3390/ijms252111832 (PMC11546233; doi:10.3390/ijms252111832)
Supplement: Supplementary file 1 [file ijms-25-11832-s001.zip › ijms-3261219-supplementary.pdf]

Supplementary information for:

Matrix Background Screening of an ssDNA Aptamer and Its Identification Against Lactopontin

Chao Zhu [1,†], Ziru Feng [1,2,†], Mengmeng Yan [1], Hongxia Du [1,*], Tengfei Li [2] and Jiangsheng Mao [1]

1 Institute of Quality Standard and Testing Technology for Agro-Products, Shandong Academy of Agricultural Sciences, Jinan 250100, China

2 College of Life Sciences and Food Engineering, Hebei University of Engineering, Handan 056038, China

* Correspondence: duhongxia@saas.ac.cn

† These authors contributed equally to this work.

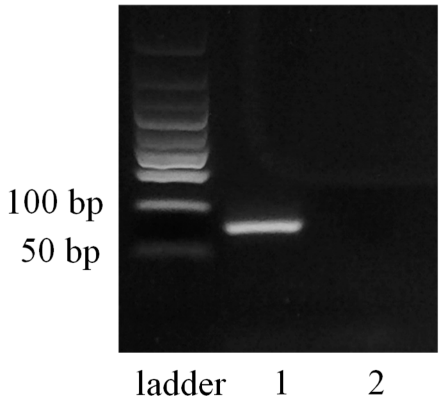

Figure S1 The clear Band 1 (Figure S1) in agarose gel electrophoresis (AGE) represented the dsDNA product of the complex after the first screening round, which was presented at the right position of about 80 bp, whereas Band 2 of blank control with no clear band. This suggested the positive result of the screening.

Table S1. The information of the Top ten candidate sequences in each approach, frequency, and their corresponding serial numbers.

Seq.	Direct approach (5`-3`)	Frequencies	Corresponding serial numbers in indirect approach
I-1	GTCAAGAAGCAACTTTAGAAGCAG CCCAGAGGTCGTGTAT	3595	3
I-2	GGAGGGCAGTCAAAAACGGGCAGC ACTCTAGTAAAGGTCG	627	40
I-3	GAGACGTGTATAGCACCAATTGAAT CAAGAAGCAGCTACC	557	251
I-4	GGGCATGGGAAGTAGGGATAGGCC GGTTTCCACCAATGAG	222	546
I-5	GCCAATTTCCGGGCACCTGGACACA GAATACTGATAGGTT	177	166
I-6	GCTGAGCGTTTCCCATATGCGCGCG GGTTTGTTTCAGCAT	173	508
I-7	TATGATCACGAAACCACATGCATTAT GTGAGGAGATGCCA	167	514
I-8	GGCGTAAAGTGATCGGTACGGGAA AGGGAAGGATGCTTAT	157	4
I-9	GCCGCACAGTCACGGCAGCTAGAT GTCGATACCGTCGGTG	135	408
I-10	GAACAGTAGAATGCACATAACCAA GAAGCAGCAGTAAACA	130	-

Seq.	Indirect (5`-3`)	Frequencies	Corresponding serial numbers in direct approach
II-1	GGTGTAGTGAGGTTGTTGCATGGG TTTACGCGTGTACGGT	27306	-
II-2	GGATCTATGTCATCACACACGGAT GGAGGAGTGCATTCGCT	2683	-
II-3	GTCAAGAAGCAACTTTAGAAGCA GCCCAGAGGTCGTGTAT	1680	1
II-4	GGCGTAAAGTGATCGGTACGGGA AAGGGAAGGATGCTTAT	1111	8
II-5	GTCAAGCGTCGGTGCCGCTCGGG GAGCCCACTAATGGATG	441	-
II-6	GGTGTAGTGAGGTTGTTGCATGGG TTTACGCGTGTACGGC	415	-
II-7	GGTGTAGTGAGGTTGTTGCATGGG CTTACGCGTGTACGGT	375	-

II-8	GGTGTAGTGAGGTTGCTGCATGGG<br>TTTACGCGTGTACGGT	373	-
II-9	GTGGTAGTTCAGGAATTCGGTAAG<br>ACGGTTGGGTCCAAGT	359	-
II-10	GGTGTAGTGAGGTCGTTGCATGGG<br>TTTACGCGTGTACGGT	339	-

Table S2. The preliminary evaluation of the binding affinity of total sequences.

**Seq.**	**I1II3**	**I2II40**	**I-3**	**I-4**	**I-5**	**II-1**	**II-2**	**II4I8**	**II-5**
Binding affinity (K_D, nM)	4.448	18.07	15.18	10.32	10.08	5.597	6.763	16.1	19.54

Table S3. Detailed interaction information of MD between BLF and Seq. C2.

Hydrogen Bonds	Hydrophobic Interactions	Pi-Pi
ALA 4-Npl…O2-A 37	PHE 8…A 37	PHE 8…A 37
CYS 9-Npl…O2-G 38		
GLY 12-Nam…Nar-G 38		
ALA 14-Npl…O2-G 68		
ALA 14-Nar…O2-T 69		
